# Current Treatment of Uveal Melanoma

**DOI:** 10.3390/cancers17091403

**Published:** 2025-04-23

**Authors:** Katie Hanratty, Gráinne Finegan, Keith D. Rochfort, Susan Kennedy

**Affiliations:** 1 School of Biotechnology, Dublin City University, Collins Avenue, Glasnevin, Dublin 9, D09 V209 Dublin, Ireland; katie.hanratty6@mail.dcu.ie (K.H.); grainne.finegan3@mail.dcu.ie (G.F.); keith.rochfort@dcu.ie (K.D.R.); 2 Research Foundation, Royal Victoria Eye and Ear Hospital, Adelaide Road, Dublin 2, D02 XK51 Dublin, Ireland; 3 Life Sciences Institute, Dublin City University, Collins Avenue, Glasnevin, Dublin 9, D09 V209 Dublin, Ireland

**Keywords:** uveal, uveal melanoma, immunotherapy, metastasis, CTLA-4, PD-1, PD-L1, TMB

## Abstract

Uveal melanoma is a rare eye malignancy with a 50% overall metastasis rate. Local control is typically successful, with low overall rates of recurrence. Survival following metastasis averages less than one year. Metastasis treatments to date have yielded poor response rates and often are seen as palliative care measures. Advanced cutaneous melanoma treatment has benefitted from the recent development of immunotherapy, in particular immune checkpoint inhibitors. Comparatively, uveal melanoma cases have not seen therapeutic benefit from these drugs. This is likely due to the difference in genetic signatures and the sun-shielded nature of uveal melanoma. The development of novel antibody modalities such as bi-specific T-cell engagers and targeted molecular therapy has benefitted outcomes for select cohorts of patients. Future avenues are currently being developed to increase the efficacy of immunotherapies for these patients. These include a combination of different therapy modalities and patient stratification based on biomarkers of sensitivity to immunotherapies and novel targeted molecular therapy.

## Simple Summary

This review examines the current treatment landscape for uveal melanoma, highlighting effective local therapies such as enucleation and plaque brachytherapy. Despite advances in medical therapeutics, the outcome for metastatic uveal melanoma has not improved significantly in the past 50 years, with immune checkpoint inhibitors showing limited success. The review discusses the role of local therapies and examines various treatment options for uveal melanoma, ranging from conventional chemotherapy and checkpoint inhibitors to newer molecularly selective T-cell therapies. Additionally, emerging strategies such as biomarker-driven patient stratification and multimodal therapies, including inhibition of the mitogen-activated extracellular signal-regulated kinase (MEK) pathway, histone deacetylase (HDAC) inhibitors, and spliceosome-targeted treatments such as splicing factor 3b subunit 1 (SF3B1) inhibitors, are discussed as potential avenues to improve patient outcomes.

## 1. Introduction

Uveal melanoma (UM) is the most common primary intraocular malignancy in adults worldwide [1]. It is characterised by melanocytic tumour growth within the uveal tract of the eye. There are an average of 9.5 cases per million of UM in Ireland and other Northern European countries each year [2]. UM represents 3–5% of all melanomas [3,4]. The average age for diagnosis is around 62 years [1].

The majority of UM patients are of Caucasian race, which is consistent with the literature on other melanoma subtypes [5,6,7]. The reason behind low rates of UM in African, Asian, and Hispanic ethnicities remains a controversial and nuanced question. Studies have theorised a link to the protective mechanisms of eumelanin against ultraviolet radiation (UVR) produced in the melanocytes of darker eye colours (more common in non-Caucasians) compared to pheomelanin, lighter eye colours (more common in Caucasians) [8,9]. However, some studies, as well as the 2018 WHO classification status [10], have refuted this claim due to the lack of support towards UVR as a factor for UM development [11].

The tumour mutational burden (TMB) is characteristically low in uveal melanoma. There are a small number of genetic mutations common to UM, including initiating mutations which do not affect prognosis, such as guanosine nucleotide-binding protein alpha-11 (GNA11), guanosine nucleotide-binding protein alpha-Q (GNAQ), cysteinyl leukotriene receptor 2 (CYSLTR2), and phospholipase C beta 4 (PLCB4) [12,13]. Mutations in BRCA1-associated protein 1 (BAP1), splicing factor 3b subunit 1 (SF3B1), initiation factor 1A X-linked (EIF1AX), and serine and arginine-rich splicing factor 2 (SRSF2) are prognostically important [12,13].

UM has overall low rates of primary tumour recurrence in patients following treatment; however, metastasis rates to distant systemic sites are as high as 50% [1]. Therapeutic approaches for advanced UM to date have been insufficient in combatting the spread of disease. New advancements in therapies are vital for metastatic uveal melanoma (mUM) patients, who have minimal effective options for treatment.

This review will focus on the pathogenesis and subsequent hallmarks of the UM disease state, the current treatments for primary and metastatic disease, as well as the recent development and future directions of immunotherapies for mUM patients.

## 2. Uveal Melanoma

### 2.1. Overview: Prognosis

**Figure 1:**
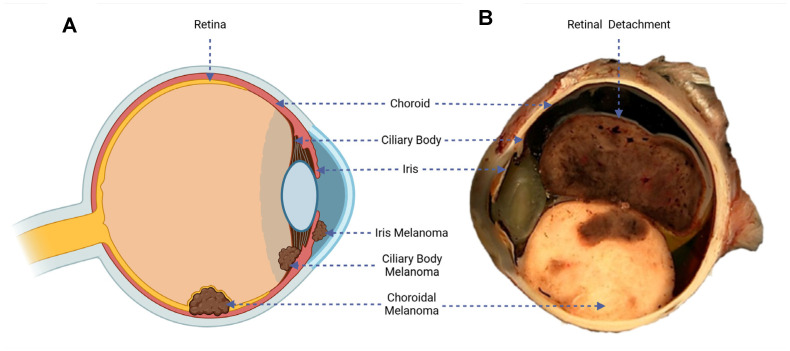
(**A**) Diagram of the eye, illustrating the three possible sites of UM development. (**B**) Macroscopic cross-section of an enucleated eye, highlighting variegated UM tumour growth and a detached retinal rim [15].

UM is a malignancy of the melanocytic cells developed within the uveal tract of the eye. These melanocytes in the eye acquire mutations over time that allow the cells to uncontrollably divide and lead to tumour development. In total, 90% of UM cases develop in the choroid, 5–8% in the ciliary body, and 2–5% in the iris (see Figure 1A) [14].

The symptoms are dependent on the area of tumour growth within the eye. Metamorphopsia (painless vision loss) is the most common symptom in UMs. Larger choroidal melanomas are associated with retinal detachment, which is associated with impaired vision (see Figure 1B). Asymmetric astigmatism is common in ciliary body melanomas. Iris melanomas are mainly associated with obvious growth or change in colour of the iris [16].

Some cases can present asymptomatically, and the disease in many cases can go undetected for some time in patients, particularly choroidal and ciliary body melanomas, where the tumour is not visible subclinically [17]. In this case, the lesion is discovered as a result of routine fundus imaging or slit lamp examination of the back of the eye at optometry appointments, where a patient will be referred to ocular oncology for investigation. In cases where this may remain undetected or treatment is neglected, patients may experience a variety of symptoms, including blindness due to retinal detachment, pain resulting from glaucoma, proptosis (bulging of the eye) due to local invasion of surrounding tissues (also known as extraocular spread), and systemic spread or metastasis of tumour cells to different organs within the body [18,19].

Diagnosis of UM has improved in accuracy throughout the years with the development of diagnostic tools and with the increased experience of ocular oncologists [20]. Diagnosis can be made through various imaging techniques such as magnetic resonance imaging (MRI), computerised tomography (CT), ocular ultrasound, or fluorescein angiography [21,22]. Fine Needle Aspirate (FNA) biopsies may be needed in more challenging cases of UM, for example, to rule out a metastatic tumour. Complications that come with this procedure include possible retinal detachment and vitreous haemorrhage [23]. In choroidal tumour cases, which clinically may represent a benign naevus, clinics often adopt a “watch-and-wait” protocol, also known as active surveillance, where treatment will be deferred until further growth is observed [23]. This is mainly because the imaging of smaller uveal melanomas is hard to distinguish from a benign choroidal naevus. These nevi only develop into melanomas in about 0.0001% of cases, so distinguishing between these lesions is important for patient care [24].

Certain molecular biomarkers can impact a patient’s prognosis and likelihood of metastasis in UM. These include tumour-suppressor BAP1 expression loss (often accompanied by monosomy 3), chromosome 8 gain, and epithelioid cell type [25,26]. Loss of BAP1 expression, which often occurs alongside partial deletion of chromosome 3, is strongly associated with a high risk of metastasis and poor survival rates due to a highly aggressive tumour [25,26,27]. Similarly, the gain of chromosome 8q is associated with reduced survival outcomes and enhanced tumour proliferation. It is also linked with immune system dysfunction, which further contributes to a worse prognosis [25,28]. Other standard prognostic tests include tumour size and cell type. Tumours of a large thickness and basal diameter have been associated with a poorer prognosis. Additionally, an epithelioid cell tumour morphology is generally a more invasive phenotype, as epithelioid cells are characterized by large nuclei, which further contribute to the aggressive nature of the tumour [25,26]. The status of these biomarkers helps clinics determine how likely a patient is to metastasise, so adequate levels of surveillance can be taken following local disease control through the techniques outlined below.

### 2.2. Current Treatments

#### 2.2.1. Treatment of Primary Uveal Melanoma

Upon diagnosis, patients have several different avenues of treatment, dependent on patient preference and characteristics of the tumour (e.g., approximate size and area).

Enucleation (removal of the eye) was the most common method of local treatment for UM until recent times [29]. The oncology team would surgically remove the whole eye from the body to prevent further local growth and systemic spread. The majority of UMs can be treated with this method and it is encouraged for cases of large tumour size.

More recently, targeted radiotherapy, also known as plaque brachytherapy, has emerged as a popular choice due to the eye-saving and possible sight-saving aspects of this treatment [30]. This may not be suitable for all UM cases, particularly in large UM tumours or where the placement of the tumour is unsuitable [29]. Brachytherapy isotopes include ruthenium 106 as well as iodine 125. This surgery involves the placement of a radioactive plaque into the socket of the eye, directly at the site of the tumour. This plaque will gradually kill the tumour cells, and is subsequently removed after radiotherapy treatment has finished. While this treatment is eye-saving, in many instances, treated eyes can experience vision loss and pain post-operation, due to increased risk of radiation retinopathy and secondary glaucoma [29].

Both treatments have comparable survival rates, and neither shows superiority in terms of tumour metastasis [31,32]. Other less common methods of treatment include proton beam radiotherapy, which uses protons as targeted irradiation instead of a plaque, and transpupillary thermotherapy, which uses infrared light to increase the temperature of the choroid to kill the tumour cells [33].

Interestingly, a phase 3 randomised trial is currently underway to evaluate the use of Belzupacap Sarotalocan (AU-011), a type of papillomavirus-like particle (VLP), in the treatment of patients with primary indeterminate lesions or small choroidal melanoma [34]. VLPs consist of recombinantly synthesized capsid proteins that assemble into a viral-like capsid, excluding viral genetic material. As anticancer agents, VLPs offer efficient delivery of drugs to tumours without relying on viral replication, reducing biosafety risks. Their mechanism involves binding of the VLPs to modified glycosaminoglycans (GAGs) present on cancer cells [35].

Additionally, a phase 2 clinical trial (NCT05907954) is investigating darovasertib (IDE196), a protein kinase C (PKC) inhibitor, as a neoadjuvant/adjuvant treatment prior to local therapy for patients with tumours harbouring GNA11 or GNAQ mutations [36,37]. These mutations, found in approximately 90% of UM patients, activate the PKC pathway, and promote tumorigenesis and metastasis. By inhibiting PKC’s, darovasertib can subsequently inhibit the proliferation of UM, induce tumour shrinkage, and reduce cell viability in mUM [37].

#### 2.2.2. Treatment of Metastatic Uveal Melanoma

Metastasis in UM occurs primarily through haematogenous dissemination, in most cases to the liver (90%), followed by the lungs, bone, and brain [38]. Unlike cutaneous melanoma, UM does not travel to lymph nodes first, as the uveal tract lacks lymphatic vessels [39].

Therapies for mUM are limited, and some treatments may not be suitable for all patients. Systemic intravenous (IV) chemotherapies, primarily alkylating agents such as fotemustine and dacarbazine [40], are less commonly used methods of treatment for mUM. This is due to their overall low efficacy of <10% [41] and significantly higher toxicological profile compared to the more modern targeted therapy approaches [42].

The first use of a liver-directed therapy approach was in 1961 by Dr. Robert K. Ausman, changing the focus of metastatic treatment for liver metastasis [43]. Table 1 presents the current mainstream hepatic-based therapies used to treat metastatic disease in UM, including their efficacy rates and limitations.

While localised treatments for primary tumours remain quite successful for UM patients, survival rates for advanced UM have remained consistently poor over time, despite the continued development of these liver-directed therapies [7,51].

The poor overall efficiency of these therapies for mUM has led to the development of more advanced approaches to targeting metastatic tumours in these patients. At present, the main therapy developed by researchers and clinicians is immunotherapy, which has shown high efficiency and increased survival rates in metastatic melanoma cases.

## 3. Current Immunotherapies in mUM

Immunotherapy is commonly used for the treatment of advanced melanoma patients. These drugs target the immune system of the patients to try to improve or increase the anti-tumour immune cell response. The two main types of immunotherapies for mUM currently in use are immune checkpoint inhibitor therapies and T-cell-directed therapies.

### 3.1. Immune Checkpoint Inhibitor Therapy

Checkpoint proteins are a class of proteins on T-cells that are crucial players in immune regulation in the body. In healthy patients, these checkpoint proteins will attach to corresponding ligands, primarily presented by antigen-presenting cells (APC) and tissue cells in response to inflammation, to ensure the downregulation of T-cell activation and cytotoxicity when necessary. This is important in the immune tolerance of the body and dampens the immune responses, to prevent damaging healthy tissues [52].

Tumour cells can often develop mechanisms to avoid being recognised by the immune system, known as immune evasion. One of these mechanisms is the expression of checkpoint protein ligands on the surfaces of the tumour cells. These ligands can attach to the checkpoint proteins on the surface of the T-cell, subsequently deactivating such, ensuring it does not recognise the tumour and provoke an immune response [53].

Immune checkpoint inhibitors (ICIs) are a class of immunotherapy drugs derived from antibodies that use this mechanism of immune evasion to their advantage. These drugs target either the checkpoint proteins expressed on the surface of T-cells or the corresponding ligand on the tumour cell. The two main checkpoints currently targeted in melanoma are cytotoxic-T-lymphocyte-associated protein 4 (CTLA-4), programmed death-protein 1 (PD-1), and programmed death-ligand 1 (PD-L1) [54].

#### 3.1.1. CTLA-4

T-cell activation can occur when a T-cell receptor (TCR) recognises a peptide expressed by the major histocompatibility complex (MHC) on an APC. Then, the CD28 molecule on the T-cell surface binds to the CD80/CD86 on an APC, which acts as a costimulatory signal for full T-cell activation [55].

**Figure 2:**
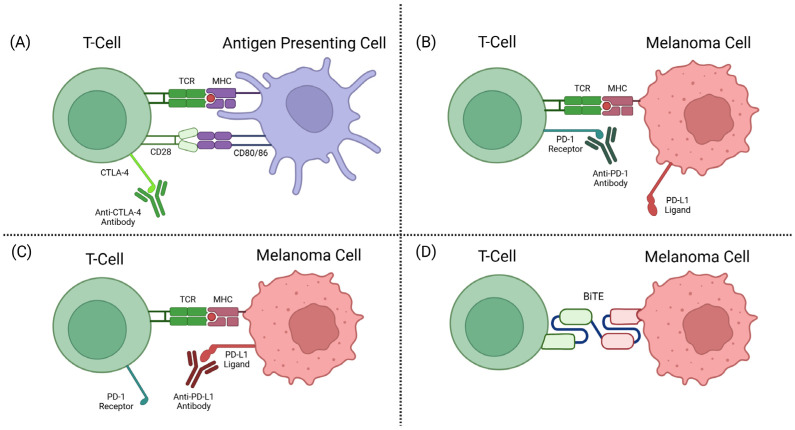
(**A**) CTLA-4 inhibitor mechanism of action (MOA). (**B**) PD-1 inhibitor MOA. (**C**) PD-L1 inhibitor MOA. (**D**) BiTE MOA [58].

The CTLA-4 receptor plays a role in immune regulation by the competitive inhibition of CD28-CD80/86 binding. CTLA-4, when bound to CD80/86, also activates intracellular signalling to inhibit proliferation and activation of T-cells [56,57]. Anti-CTLA-4 ICIs target the T-cell-expressed CTLA-4 receptor that plays a role in T-cell inhibition, allowing the T-cell to remain activated and target the tumour cells (see Figure 2A).

Ipilimumab is an anti-CTLA-4 humanised monoclonal antibody (mAb) most commonly used for melanoma patients. Ipilimumab was the first FDA-approved ICI for advanced melanoma patients, with response rates of about 10–20% [59].Despite the low response rates, this was a huge step in melanoma therapies at the time.

#### 3.1.2. PD-1 and PDL-1 Inhibitors

In healthy individuals, PD-1 and PD-L1 interactions help to deactivate T-cells and reduce proliferation in later stages of T-cell activation. Tumours have adopted mechanisms of expressing PD-L1 to become unrecognisable as tumours by T-cells and subsequently deactivate these immune defences from killing the tumour cells [53]. Anti-PD-1 antibody drugs are a class of ICIs targeting the checkpoint protein PD-1 on a T-cell surface, or its corresponding ligand PD-L1. This prevents the interaction between PD-1 and PD-L1, as well as the subsequent deactivation mechanisms (see Figure 2B,C).

In 2014, the FDA approved nivolumab and pembrolizumab, two mAbs targeting PD-1 for unresectable or metastatic melanoma [60,61]. This therapy has been highly effective in advanced cutaneous melanoma cases, where overall response rates can reach up to ~45% [62]. However, much lower response rates are seen from UM tumours across many retrospective studies and clinical trials, averaging around 0–10% [63].

Combination therapies are now a common mode of administration for ICI therapies. This involves the administration of an anti-PD-1/anti-PD-L1 antibody combined with an anti-CTLA-4 antibody to maximise the effect, mainly a combination of ipilimumab and nivolumab [64]. This has seen even higher efficacy rates in advanced melanoma patients (58% ORR in phase 3 clinical trial) [65]. However, the increase in adverse effects compared to monotherapy makes this therapy unsuitable for immunocompromised or severely ill patients.

Despite ICI’s revolutionising the treatment of cutaneous metastatic melanoma cases, the overall responses for UM have been poor. This has been theorised to be attributed to UM’s low mutational burden average (1.1 Mut/Mb) compared to cutaneous melanomas (18 Mut/Mb) [26]. A study by Dousset et. al [66] showed a positive correlation between low TMB and poor responses to ICI therapies [67]. The characteristically low TMB rate of UM is likely related to the sun-shielded location of UM development: UM is an intraocular malignancy, partially blocked from ultraviolet radiation (UVR), unlike skin melanomas that are much more exposed to UVR [68]. High exposure to UVR results in higher rates of UV-mediated DNA mutations, and tumours are thus more likely to express immune-modulation mechanisms and neoantigen production [69].

The poor response rates seen in mUM in comparison to other forms of melanoma have gained traction within immune-oncology groups, to develop more effective immunological approaches in mUM management.

### 3.2. T-Cell Direction Therapy

The main immunotherapy that has shown promising results in UM patients is Tebentafusp (TEBE). TEBE is the first engineered soluble T-cell receptor (TCR) that acts as a bispecific T-cell engager (BiTE) [70]. Developed by Immunocore, TEBE was approved by the FDA in January 2022, under the brand name Kimmtrak [71].

**Figure 3:**
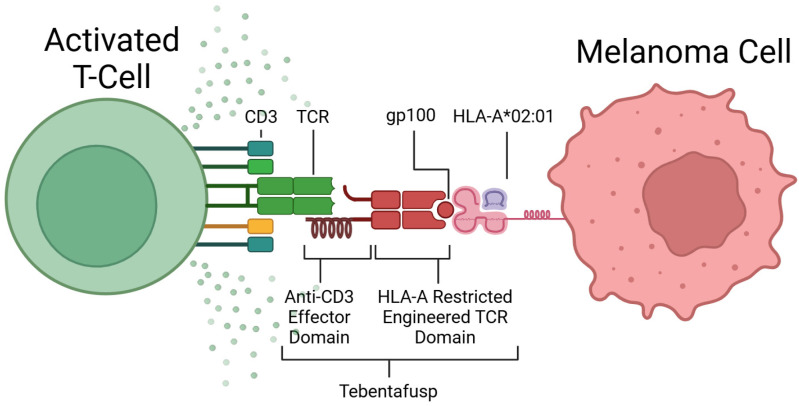
Mechanism of action of Tebentafusp. Created in BioRender [74].

The discovery of TCR recognition of glycoprotein 100 (gp100) began the development of this form of therapy [72]. TEBE’s TCR binds to gp100, located on the surface of melanoma cells, and its anti-CD3 domain binds to CD3, located on all T-cells (see Figure 3) [68]. This bispecific binding brings T-cells into close contact with melanoma cells and creates a bridge between the two cells, where the T-cell recognises the tumour cell and induces apoptosis (see Figure 2D) [73].

TEBE is used in the treatment of mUM patients who are Human Leukocyte Antigen-A*02:01 (HLA-A*02:01)-positive, which consists of around 50% of the Caucasian population [70,75]. HLA-A*02:01 is a specific variant of the human leukocyte antigen (HLA) expressed as a cellular marker on melanoma cells. Treatment is not suitable for patients without this selective biomarker, as TEBE is specific for both the HLA-A*02:01 molecule and gp100, so it will not bind if gp100 is expressed on a different variant of HLA.

TEBE’s trials to date have shown impressive results in mUM patients. In a phase 1/2 clinical trial involving eighteen mUM patients, conducted over one year, three showed partial response, eight showed stable disease, and seven showed progressive disease [76]. The study also interrogated the tumour microenvironment and immune system responses through various biomarker analyses, such as cytokine CXCL10 and CXCR3 T-cell concentration levels. They found an overall increase in T-cell markers from immunohistochemical staining of pre-treatment and post-treatment tumour biopsies.

A much larger phase 3 clinical trial including a control group showed overall survival (OS) at one year of 59% in the control vs. 73% in the TEBE-treated group, along with an increase in disease-free survival [70]. Many other clinical trials have supported the evidence that TEBE is an efficient treatment option for suitable patients.

Despite TEBE’s efficiency in response to mUM, its limitation to only HLA-A*02:01-positive patients is a valid concern for clinics. More universal treatment strategies are needed to increase the therapeutic options for mUM patients who do not qualify for TEBE.

### 3.3. Clinical Trials

Table 2 presents the immunotherapy and T-cell therapy treatments for mUM that are currently undergoing phase II or phase III clinical trials.

## 4. Future Directions

To combat the efficacy issues with current treatments, researchers have been developing new strategies that can help close the gap in therapies for mUM. The three main directions focused on in this review are patient stratification, multimodal therapeutics, and emerging targeted therapeutics.

### 4.1. Selective Biomarkers: Patient Stratification

As previously described, across the UM population, there is a poor response rate to ICIs compared to their cutaneous counterpart [67]. This is likely down to the lower TMB rates associated with uveal melanoma, where the tumour is less likely to produce the necessary immune evasion mutations that are targeted in ICI therapies [67].

However, a subset of patients with UM with high TMB has previously been identified in the literature [79,80,81]. The patient subsets of interest described by Johansson et al. (2020) have either a unique germline mutation of Methyl-CpG Binding Domain 4 (MBD4) or UVR damage, a signature seen in iris melanoma due to tumour location [79]. These two characteristics cause the tumours to become hypermutated and increase in mutations per megabase (mut/Mb).

As previously described, high TMB is a biomarker of an increased affinity for ICI therapies, and patients with this tumour signature are potential candidates for this treatment. By analysing TMB status in patients, focusing on patients with MBD4 mutations or UVR signatures, oncologists can potentially stratify patients more likely to benefit from these treatments and, therefore, achieve better overall patient outcomes. However, further studies are needed to validate and develop these findings.

### 4.2. Multimodal Therapies

Another avenue currently being trialled for increasing response rates of mUM to immunotherapies is to combine them with other forms of systemic and liver-directed therapies.

In a retrospective analysis of dual-treated patients at the University Medical Centre Hamburg-Eppendorf [82], a variety of historically combined therapies, exclusive to ICI combinations, were reviewed. This included dual-ICI with TACE and surgical resection. They found that overall survival (OS) rates increased with all forms of combined ICI and other modes of therapy (22.47 months versus 11.37 months). However, they did not compare the different therapies with each other to find the combination that most significantly improves OS.

Some specific therapies are currently being evaluated for their efficacy in combination with ICIs. These include hepatic perfusion with melphalan, SIRT, TACE, and immunoembolization [83]. A Phase 1 trial of percutaneous hepatic perfusion with melphalan in a small cohort of patients showed promising results, where all patients had either stable disease, partial, or full responses [84]. Further, a retrospective study of a cohort of SIRT-treated mUM, and combined SIRT- and ICI-treated mUM showed greatly increased OS in the combined treatment group (49.6 months) versus the SIRT group (13.6 months) [85].

The combination of different modes of therapy with immunotherapy has shown promising results compared to immunotherapy alone and may be a potential avenue for increasing the efficacy of immunotherapy in mUM. However, there is a need for further studies and clinical trial results that can back up these results.

### 4.3. Emerging Targeted Therapies

Emerging therapies for mUM focus on novel targeted therapeutic strategies beyond current systemic and liver-directed therapies. Three targeted therapeutics of particular interest for mUM are mitogen-activated extracellular signal-regulated kinase (MEK) pathway inhibitors, histone deacetylase inhibitors, and spliceosome-targeted therapies.

GNA11/GNAQ somatic activating mutations occur in up to 90% of UM cases, which subsequently activates the MEK pathway. As a result, MEK inhibitors, such as selumetinib and trametinib, are currently being investigated as targeted therapies for mUM [86,87]. MEK inhibitors have shown success in some clinical trials but unfortunately have not been seen to improve OS due to acquired resistance [88]. To counteract this by blocking other cell signalling pathways, c-mesenchymal-epithelial transition factor (c-MET) and phosphoinositide 3-kinase (PI3K) inhibitors could be a solution to MEK inhibition resistance [88].

Roginolisib (IOA-244) is the first highly selective, allosteric modulator of Phosphoinositide 3-kinase delta (PI3Kδ) [89,90]. It inhibits PI3Kδ through non-adenosine triphosphate (ATP) competitive binding to the C-terminal region, effectively regulating its activity, while avoiding the side effects typically associated with other PI3K inhibitors [91]. Roginolisib is currently being investigated and has shown promise for overcoming resistance mechanisms in MEK-targeted therapies.

The combination of darovasertib (IDE196), a PKC inhibitor, with crizotinib, a mesenchymal-epithelial transition factor (MET) inhibitor, is another emerging strategy. This combination is being investigated as a first-line treatment in HLA-A*02:01-negative patients with mUM in a phase 2/3 multi-arm, multi-stage clinical trial (NCT05987332) [92]. The study aims to further evaluate the effectiveness of combining darovasertib and crizotinib to target GNA11 and GNAQ signalling pathways to improve clinical outcomes in patients with HLA-A*02:01-negative mUM [92].

Additionally, histone deacetylase (HDAC) inhibitors have demonstrated efficacy in reducing tumour growth in vivo. Histones are simple proteins that package DNA into structural units called nucleosomes, which organize DNA in the nucleus and play a crucial role in gene regulation [93]. By inhibiting the acetylation of histones, these agents may reverse the effects of BAP1 loss, leading to a less aggressive tumour, and potentially restoring normal gene expression [93].

The spliceosome is another promising area of research, which acts as a potential antitumoural target. The spliceosome is a large RNA–protein complex which catalyses the removal of introns from nuclear pre-mRNA. SF3B1 is a pre-mRNA splicing target that, when mutated, is a common metastatic driver in UM. Spliceostatin A and sudemycin are both examples of SF3B1 inhibitors [94]. Their mechanism of action disrupts splicing processes, leading to exon skipping or intron retention, and increasing alternative splicing patterns [94]. This SF3B1 inhibition could potentially cause an SF3B1-mutated tumour to become less aggressive in nature through defective RNA transcript production and subsequent nonsense-mediated decay activation [94].

## 5. Conclusions

Despite much improved local control, the rates of systemic metastasis of UM remain at 50% on average. While immunotherapies like ICIs have transformed the outcome of cutaneous melanoma cases, the same cannot be said for UM. Many studies explain this disparity by the differences in the genetic profiles of these tumours, particularly TMB. Despite ICI’s low effectiveness in UM compared to cutaneous melanoma, these therapies are currently in use for mUM cases. Protein engineering has facilitated the development of novel immunotherapies and molecular therapies for mUM, including the significant development of TEBE. More recent developments for increasing immunotherapy effectiveness include the stratification of mUM patients by genetic profiles and UVR signatures for increased ICI therapeutic benefits, multimodal therapy strategies, and emerging targeted therapies for metastatic tumours. These future directions require increased studies and development before implementation in clinical settings, but have shown early promising results. There remains a clear path for further developments and refinements of immunotherapy for mUM to continue to advance treatments and patient care worldwide.

## Figures and Tables

**Table 1:** Mainstream liver-directed therapies for mUM patients: limitations and efficacy (excl. immunotherapy).

Therapy	Mechanism	Efficacy	Limitations	Source(s)
Partial hepatectomy	Surgical resection of metastatic lesions.	Moderate (14–27 months MS)	Only compatible with low liver involvement and well-circumscribed lesions (2–7% of cases).	[44,45]
Hepatic perfusion and melphalan (isolated/percutaneous)	Local high-dose chemotherapy.	Low (ORR ~20–40%)	Hepatic toxicity; invasive and unrepeatable procedure (isolated).	[46,47]
Transarterial chemoembolization (TACE)	Local chemotherapy and embolization of the hepatic artery.	Low (~20% ORR, ~15 months MS)	Only compatible with non-diffuse lesions that obtain blood supply solely from the hepatic artery.	[48,49]
Selective internal radiotherapy (SIRT)	Local radiotherapy (yttrium beads) into the hepatic artery.	Low (~12.3 months OS)	Unsuitable for patients with poor liver function or diffuse lesions.	[50]

ORR = overall response rate; MS = median survival; OS = overall survival.

**Table 2:** Current phase II/III clinical trials in metastatic uveal melanoma immunotherapy treatments [77].

Clinical Trial Reference No.	Treatment	Phase	Status	Note
NCT02697630	Pembrolizumab (anti-PD-1) + Entinostat (HDAC inhibitor)	II	Completed	Benefits only a subset of patients [78]
NCT03472586	Immunoembolization + Ipilimumab (anti-CTLA-4) + nivolumab (anti-PD-1)	II	Completed	Stable disease only (57%)
NCT04552223	Nivolumab (anti-PD1) Plus Relatlimab (anti-LAG-3)	II	Active, not recruiting	ORR 7.7%
NCT05542342	Sitravatinib (mTKI) and Tislelizumab (anti-PD-1)	II	Active, not recruiting	
NCT05077280	Stereotactic Body Radiotherapy + Nivolumab (anti-PD1) + Relatlimab (anti-LAG-3)	II	Active, not recruiting	
NCT05308901	Lenvatinib (mTKI) + Pembrolizumab (anti-PD1)	II	Active, not recruiting	
NCT06519266	PHP + Ipilimumab (anti-CTLA-4) and Nivolumab (anti-PD-1)	III	Recruiting	
NCT06121180	Cemiplimab (anti-PD1) + Ziv-Aflibercept (anti-VEGF)	II	Recruiting	
NCT06581406	RP2 (oncolytic immunotherapy) + Nivolumab (anti-PD-1)	II	Recruiting	
NCT05524935	Olaparib + Pembrolizumab (anti-PD-1)	II	Recruiting	
NCT03467516	Tumour Infiltrating Lymphocytes	II	Recruiting	
NCT06246149	Adjuvant Therapeutic: Tebentafusp (IMCgp100)	III	Recruiting	

LAG3—lymphocyte Activation Gene 3, mTKI—multitargeted tyrosine kinase inhibitors, PHP—percutaneous hepatic perfusion, VEGF—vascular endothelial growth factor.

## Data Availability

No new data were created or analysed in this study.

## Abbreviations

The following abbreviations are used in this manuscript:

APC	Antigen-Presenting Cell
ATP	Adenosine TriPhosphate
BAP1	BRCA1-associated protein 1
BiTE	Bispecific T-cell Engager
CD	Cluster of Differentiation
c-MET	c-Mesenchymal-Epithelial Transition Factor
CT	Computerised Tomography
CTLA-4	Cytotoxic T-Lymphocyte Associated Protein 4
CYSLTR2	Cysteinyl Leukotriene Receptor 2
CXCL	Chemokine (C-X-C motif) Ligand
CXCR3	C-X-C Chemokine Receptor 3
EIF1AX	Initiation Factor 1A X-linked
FDA	Food and Drug Administration
FNA	Fine Needle Aspiration
GAGs	Glycosaminoglycans
GNA11	Guanosine Nucleotide-Binding Protein Alpha-11
GNAQ	Guanosine Nucleotide-Binding Protein Alpha-Q Gene
gp100	Glycoprotein 100
HDAC	Histone Deacetylase
HLA	Human Leukocyte Antigen
HR	Hazard Ratio
ICI	Immune Checkpoint Inhibitor
IV	Intravenous
LAG3	Lymphocyte Activation Gene 3
mAb	Monoclonal Antibody
MBD4	Methyl-CpG Binding Domain Protein 4
MEK	Mitogen-Activated Extracellular Signal-Regulated Kinase
MET	Mesenchymal-Epithelial Transition Factor
MHC	Major Histocompatibility Complex
MRI	Magnetic Resonance Imaging
MS	Median Survival
mTKI	Multitargeted Tyrosine Kinase Inhibitors
mUM	Metastatic Uveal Melanoma
OS	Overall Survival
PD-1	Programmed Cell Death Protein 1
PD-L1	Programmed Death-Ligand 1
PHP	Percutaneous Hepatic Perfusion
PI3K	Phosphoinositide 3-Kinase
PI3Kδ	Phosphoinositide 3-kinase delta
PKC	Protein Kinase C
PLCB4	Phospholipase C Beta 4 (PLCB4)
PV	Papillomaviruses
SF3B1	Splicing Factor 3B Subunit 1
SIRT	Selective Internal Radiotherapy
SRSF2	Serine and Arginine Rich Splicing Factor 2
TACE	Transarterial Chemoembolization
TCR	T-cell Receptor
TEBE	Tebentafusp
TMB	Tumour Mutational Burden
UM	Uveal Melanoma
UVR	Ultraviolet Radiation
VEGF	Vascular Endothelial Growth Factor
VLP	Viral-Like Particles
